# Stress and coping in the face of COVID-19: a qualitative inquiry into early pandemic experiences and psychological well-being of health workers in Burkina Faso, Senegal and The Gambia

**DOI:** 10.1093/heapol/czad023

**Published:** 2023-04-10

**Authors:** Julia Lohmann, Marème Diallo, Manuela De Allegri, Jean-Louis Koulidiati, Melisa Martinez-Alvarez

**Affiliations:** Department of Global Health and Development, London School of Hygiene & Tropical Medicine, 15-17 Tavistock Place, London WC1H 9SH, UK; Heidelberg Institute of Global Health, University Hospital and Medical Faculty, Heidelberg University, Im Neuenheimer Feld 130.3, Heidelberg 69120, Germany; MRC Unit The Gambia at the London School of Hygiene & Tropical Medicine, Atlantic Boulevard, Fajara, PO Box 273, Banjul, The Gambia; Institut de Recherche en Santé, de Surveillance Epidémiologique et de Formation, 4 Rue 2 D1 Pole Urbain de Diamniado BP 7325, Dakar, Sénégal; Heidelberg Institute of Global Health, University Hospital and Medical Faculty, Heidelberg University, Im Neuenheimer Feld 130.3, Heidelberg 69120, Germany; Institut Supérieur des Sciences de la Santé, Université Nazi Boni, 01 BP 1091, Bobo-Dioulasso, Burkina Faso; MRC Unit The Gambia at the London School of Hygiene & Tropical Medicine, Atlantic Boulevard, Fajara, PO Box 273, Banjul, The Gambia; Institut de Recherche en Santé, de Surveillance Epidémiologique et de Formation, 4 Rue 2 D1 Pole Urbain de Diamniado BP 7325, Dakar, Sénégal; Université Cheikh Anta Diop, Fann Campus, Dakar BP 5005, Sénégal

**Keywords:** COVID-19, health workers, experiences, coping, well-being, Burkina Faso, Senegal, The Gambia

## Abstract

COVID-19 represented an unprecedented challenge for health workers around the world, resulting in strong concerns about impacts on their psychological well-being. To inform on-going support and future preparedness activities, this study documented health workers’ experiences, well-being and coping throughout the first wave of the pandemic, in Burkina Faso, Senegal and The Gambia. We collected data from 68 primarily clinical staff from the COVID-19 treatment, maternity and emergency departments in 13 purposely hospitals and laboratories across the three countries. Following in-depth interviews via Zoom (mid-May to September 2020), we regularly followed up via WhatsApp until the end of 2020. We used a mixed deductive and inductive coding approach and a framework matrix to organize and analyse the material. All respondents initially assessed the situation as stressful and threatening. Major emotional reactions included fear of own infection, fear of being a risk to loved ones, guilt, compassion, and anxiety regarding the future. Many suffered from feeling left alone with the emerging crisis and feeling unvalued and unappreciated, particularly by their governments and ministries of health. Conversely, health workers drew much strength from support and valuation by direct supervisors and team members and, in part, also by patients, friends and family. We observed important heterogeneity between places of work and individual backgrounds. Respondents coped with the situation in various ways, particularly with strategies to manage adverse emotions, to minimize infection risk, to fortify health and to find meaning in the adverse circumstances. Coping strategies were primarily grounded in own resources rather than institutional support. Over time, the situation normalized and fears diminished for most respondents. With a view towards emergency preparedness, our findings underline the value of participation and transparent communication, institutional support and routine training to foster health workers’ psychological preparedness, coping skill set and resilience more generally.

Key messages

- Health workers in Burkina Faso, Senegal and The Gambia experienced the initial weeks of the COVID-19 pandemic as a time of extreme stress but showed remarkable resilience, adjusting quickly to the new reality.
- They experienced a range of initially primarily negative but increasingly positive emotions.
- In coping with difficult emotional experiences, they had to rely primarily on their own resources.
- In preparing for future emergencies, our findings underline the value of participation and transparent communication, institutional support and routine training to foster health workers’ psychological preparedness, coping skill set and resilience more generally.

## Introduction

As the COVID-19 pandemic made its way around the globe, pictures of overwhelmed health workers generated awareness of the difficulties they face on a daily basis. Indeed, the pandemic added a significant burden to this already demanding profession. Even prior to the pandemic, health workers suffered from high rates of mental illness and poor psychological well-being (McVicar, 2003; Aiken *et al*., 2012; Lemaire and Wallace, 2017), resulting in adverse consequences for not only affected individuals but also patients and health systems in general (Davey *et al.*, 2009; Aiken *et al.*, 2012; Hall *et al.*, 2016; Wilkinson *et al.*, 2017).

The pandemic generated an unprecedented amount of research on health workers’ experiences and their emotional impact. As during prior epidemics (Billings *et al.*, 2021; Busch *et al.*, 2021), the COVID-19 pandemic introduced or exacerbated many health-care workplace challenges, concerns for one’s physical well-being and a host of interpersonal issues (Agyei *et al.*, 2021; Xu *et al.*, 2021; Chemali *et al.*, 2022). Studies suggest an elevated prevalence of mental health issues among health workers particularly during the first months of the pandemic. A meta-review conducted in early 2021 estimated a global prevalence of psychophysiological stress of 38% and of psychopathology (i.e. anxiety, depression and post-traumatic stress disorder) of 26% (de Sousa *et al.*, 2021). Simultaneously, however, the emerging literature reveals health workers’ remarkable resilience. Agyei *et al.* (2021) synthesized qualitative research published early in the pandemic, concluding that after an initial period of chaos, health workers in various contexts transitioned to thriving, notwithstanding persisting challenges.

The surge in health worker-focused research during the pandemic has mostly excluded sub-Saharan Africa (SSA), with the exception of South Africa, Nigeria and Ethiopia, for which pre-pandemic research on health worker well-being exists (Lohmann *et al*., 2022). This is interesting given strong initial concerns about how the pandemic would overwhelm the weak health systems in most SSA countries (Martinez-Alvarez *et al.*, 2020).

Particularly rare is research exploring health workers’ lived experiences and perceived psychosocial impacts in sub-Saharan African contexts. Available studies describe challenging working conditions in the early days of the pandemic, leaving health workers vulnerable to COVID-19 infection and other adverse mental health, social and economic consequences (e.g. Okediran *et al.*, 2020; Ilesanmi *et al.*, 2021; Kayiga *et al.*, 2021; Semaan *et al.*, 2022). Specific challenges reported across settings include a lack of COVID-specific knowledge, appropriate personal protective equipment (PPE) and political and management support. Additionally, long work hours under physically difficult conditions, patient-related challenges and issues related to service reorganization and compensation were noted. Studies explicitly exploring psychosocial impacts noted stigmatization, fear, distress and fatigue (e.g. Sougou *et al.*, 2020; Kwaghe *et al.*, 2021; Phiri *et al.*, 2022). Interestingly, available studies narrate only negative experiences associated with the pandemic, in slight contrast to the wider international literature (Agyei *et al.*, 2021) as well as the literature on prior pandemics (Billings *et al.*, 2021), raising the question as to whether this is an effect of research timing or whether experiences in SSA were indeed substantially different from those elsewhere in the world. Available studies from SSA also do not discuss how health workers coped with their adverse experiences. This is with the exception of Okediran *et al.* (2020), who found that in Nigeria, health workers relied almost exclusively on their own resources to deal with practical challenges and emotional difficulties while institutionalized support was largely absent, similar to previous experiences in resource-limited settings (Witter *et al.*, 2017).

With a view towards future emergency preparedness, research on how SSA health workers’ experiences developed over time and how health workers coped to preserve their health and well-being is therefore of key importance. Our study adds such evidence from Burkina Faso, Senegal and The Gambia, three low-income countries in West Africa. We present the findings of a qualitative longitudinal study conducted over the first nine months of the pandemic. Methodologically, we demonstrate the feasibility and value of performing in-depth qualitative research remotely.

## Methods

### Context

Burkina Faso, Senegal and The Gambia are low-income countries with populations of 20.9 million, 16.7 million and 2.4 million, respectively (World Bank, 2020). Progress towards achieving universal health coverage in these countries is hindered by underfunding and generally weak public health systems, resulting in a high burden of morbidity and mortality (WHO, 2018). Key challenges include insufficient availability of health-care personnel particularly in rural areas, substandard infrastructure, recurrent stockouts of drugs and other important resources and various inefficiencies within highly bureaucratic health systems and administrations.

### Study design

**Figure 1.**
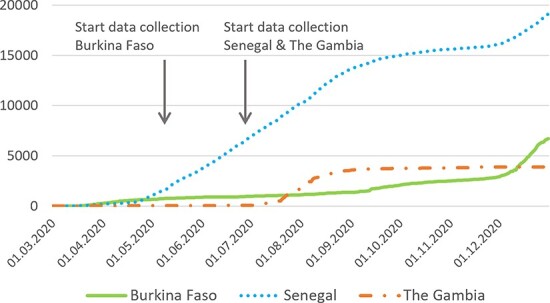
Data collection in relation to cumulative confirmed COVID-19 cases

Legend: Figure produced with data from Our World in Data (2022)

We conducted a partly retrospective, partly prospective qualitative study to capture health workers’ lived experiences during the first nine months of the pandemic. Figure 1 shows the development of the pandemic in the study countries over the course of 2020, as well as the respective start of data collection.

### Sampling

We chose Burkina Faso, Senegal and The Gambia based on our team’s previous geographic research focus. In choosing study sites and defining the sampling strategy, we assumed that beyond the state of the pandemic, health workers’ experiences would primarily differ by whether they were directly involved in the treatment of COVID-19 patients or indirectly affected by continuing routine practice under pandemic conditions, as well as by the general level of workplace preparedness. Our purposive three-step approach to sampling reflects this hypothesis. In each country, we selected institutions from each of the following three health facility categories: (1) main COVID-19 treatment centres, the primary recipients of early COVID-19 cases and preparedness activities; (2) peripheral hospitals without early exposure to COVID-19, but likely to receive patients as the outbreak unfolded and (3) laboratories engaged in collecting and testing samples. From each hospital, we then sampled 3–5 health workers from the COVID-19, maternity and emergency departments, respectively, as well as biologists and technicians from each laboratory. In hospitals, we only considered clinical staff (medical doctors, nurses, midwives and other fully trained mid-level clinical staff) as well as hygienists and stretch-bearers given their specific role in dealing with COVID-19 patients. In each institution and department, we advertised the study and asked for volunteers. To identify any biases associated with this approach, we interviewed hospital managers and members of the psychological support teams posted in many sites in the course of the study, presenting emerging study findings and asking for confirmation or qualification. Key informants confirmed that participant narratives corresponded to their perception of health worker experiences more broadly.

### Data collection

We started data collection in each country as soon as the necessary approvals were in place (Figure 1). Data were collected by the first and second authors as well as a research assistant in The Gambia, who regularly debriefed and peer supervised. Interviews were conducted in French in Burkina Faso, in French and Wolof in Senegal and in English in The Gambia. Following informed consent, we conducted an initial interview via Zoom or phone, using a semi-structured interview guide, to understand participants’ backgrounds and to retrospectively capture their work experiences during the pandemic, associated emotions and coping strategies up to date. In one hospital in Senegal, initial interviews were conducted face-to-face in line with participant preferences. Interviews were recorded and verbatim transcribed for analysis. Interviews conducted in Wolof were translated to French. Until the end of 2020, we then regularly contacted participants via WhatsApp—initially twice a week and then every 3–4 weeks as notable developments decreased—asking them for updates on their experiences and associated feelings. Updates received as voice messages were also verbatim transcribed for analysis.

We were initially unsure how comfortable participants would be with the rather impersonal remote way in which we asked them to share their experiences and inner feelings with us, a team of interviewers composed of a female Senegalese sociologist, a female German psychologist, and a male Gambian data collection professional, all highly familiar with the respective research contexts and experienced in conducting sensitive qualitative interviews. We took great care to build relationships and trust in confidentiality throughout data collection. Our concerns quickly dissipated as feedback collected through an anonymous survey several weeks into data collection confirmed high levels of appreciation of the opportunity to narrate experiences. Many participants hinted that the virtual environment helped rather than hindered sharing feelings and opinions with neutral outsiders.

### Analytical framework

We used Lazarus and Folkman’s (Lazarus and Folkman, 1984) Transactional Theory of Stress (TTS) in its revised form (Folkman and Moskowitz, 2004) to organize and interpret the material. The TTS posits that individuals are in constant appraisal of their environment, experience emotions in consequence, and initiate coping strategies to manage negative emotions potentially harmful to their well-being. Stress results if environmental influences harmful to well-being exceed the individual’s ability to cope with them. Consequently, ‘cognitive appraisal’ (individuals’ evaluations of the extent to which the situation is potentially harmful to them) and ‘coping’ (cognitive or behavioural efforts to overcome, manage, or tolerate the stressful situation) constitute the two key processes determining the extent to which a person experiences negative emotions (‘stress’) resulting from their circumstances. The TSS differentiates ‘emotion-focused coping’ (dealing with the emotions that result from the stressful situation), ‘problem-focused coping’ (attempts to change the stressful situation or environment), ‘meaning-focused coping’ (actively finding sense in overwhelmingly stressful and uncontrollable situations where other coping strategies have been unsuccessful), and ‘future-oriented coping’ (behaviour or cognition directed at anticipated stressors, before they occur).

### Data analysis

Data analysis was led by the first and second authors and triangulated by the other authors. The transcribed material was coded using the key elements of the TSS as a deductive starting point for the codebook, which was then inductively populated with sub-themes emerging from the data. Multiple analysts coded the same set of initial transcripts and discussed their results to agree on a common codebook, which the main analysts then applied to the rest of the material. Finally, the coded material was summarized into a framework matrix to support the exploration of participants’ cognitive appraisal, emotional experiences, and coping. In analysing the material, we first focused on common themes and then explored heterogeneity in the data.

## Results

We start with a description of participant characteristics, followed by a summary of health workers’ overall appraisal of the evolving situation as a potential threat to their well-being. We then outline the coping strategies health workers applied in dealing with their stressful circumstances, before describing the various resulting emotional experiences narrated by the study participants as the pandemic unfolded.

### Participant characteristics

We conducted 68 interviews across 12 institutions (10 public and 2 private) in the three countries as shown in Table 1. Participants were on average 35 years old (span: 24–63) and had 10 years of work experience (span: 1–37). Four out of five resided with family members at the time of data collection. In addition, we spoke to six managers within the ministries of health and participating hospitals as well as to four members of psychological support teams. Resulting data were used for triangulation purposes only and are not reported.

**Table 1.** Participant characteristics

	*n*	%
**Country**		
Burkina Faso	27	40
Senegal	20	29
The Gambia	21	31
**Service**		
COVID-19	34	50
Maternity	14	20
Emergency	10	15
Laboratory	10	15
**Cadre**		
Medical doctor	18	27
Nurse	26	38
Midwife	9	13
Biologist	7	10
Other	8	12
**Gender**		
Female	27	40
Male	41	60

### Appraisal

Participants across countries and settings initially assessed the pandemic situation as stressful and as a potential threat to their well-being (see Table 2 for illustrative participant quotes). Most participants were initially unsure how well they would cope with the situation, both technically and psychologically, describing it as something entirely unfamiliar. Only a handful of health workers—mainly with backgrounds or interest in infectious diseases—appraised the situation as a welcome challenge and opportunity for learning, although all were worried about how their country and health system would cope.

**Table 2.** Illustrative participant quotes—Appraisal and Coping

Main theme	Sub-theme	Illustrative quote(s)
Appraisal	Initial overall appraisal of the situation	At the time the disease was not well identified, we did not know the symptoms well; they went in all the directions. So the ‘psychosis’^a^ started to settle in our department. We were walking blindly. We did not know how to dance this dance. It was very complicated. There was much pressure, even if people didn’t express it verbally, you could feel it (male medical doctor, emergency, Senegal).
	Overall appraisal of the situation over time	At the moment, I can say that the ‘psychosis’^a^ has passed. At the moment, it is fine. It’s still a bit stressful, but we’re managing to get through it at the moment, compared to the beginning (male nurse, COVID-19, Burkina Faso).
Coping	Problem-focused coping	In private life it was a bit difficult; there were steps you had to take that were almost impossible. How do you create distance at home? (Male nurse, maternity, Burkina Faso) I exercise a lot. I know it’s good in order to boost my immune system. […] Sometimes I just lie down and sleep in my free time and then when I wake up I feel fresh (male biologist, laboratory, The Gambia). We don’t spend that much time with patients, because the longer you stay with the patient the more likely that you may be infected too (male nurse, COVID-19, The Gambia).
	Emotion-focused coping	We did not have a psychologist, but we were psychologist ourselves, we supported each other (female nurse, COVID-19, Senegal). My best therapy is to talk to the family in the evening for at least one or two hours, I tell them what’s going on, my wife cheers me up, I talk to the children, it makes me feel better and the next day I try to get back on track (male doctor, COVID-19, Burkina Faso). They also sent a psychiatrist every day to come and meet with us, to give us a moral boost (male nurse, COVID-19, Burkina Faso). I think is my faith that helps me, I always say Allah will help me, let me do the right thing helping people with clean heart, and God will take care of the rest (female nurse, maternity, The Gambia).
	Meaning-focused coping	The country needs me and if I leave and someone else leaves, there won’t be anyone left here and then everyone will get the virus. Sometimes you have to sacrifice yourself (female hygienist, COVID-19, Senegal).
	Future-oriented coping	[My wife, who is also a doctor, and I] had a discussion and we said, there’s a reasonable chance that we’ll see a lot of COVID-19, and therefore there’s a reasonable chance that we would acquire it. We kind of weighed up our chances and thought to ourselves that actually, our chances of becoming seriously unwell were really very small, and therefore we weren’t too anxious about personally getting COVID-19 (male doctor, COVID-19, The Gambia).

a Several French-speaking respondents spoke about ‘la psychose’ when referring to widespread sentiments in the early days of the pandemic. The term is not meant to refer to the respective psychiatric syndrome.

Over time, most participants reported that the situation normalized for them. Participants explained how a combination of factors related to reduced uncertainty, improved skills and routine and improved working conditions led to perceived improved ability to cope with the potential threat of the pandemic and its consequences. At the same time, new stressors appeared as the pandemic progressed, shifting psychological burden from fears of infection to frustration with working conditions and related feelings of lack of appreciation and support. While most participants experienced negative sentiments over time, many also described positive emotions emerging as time progressed. These included a strengthened sense of collegiality at work, pride of being able to help their country, being appreciated and valued for one’s work and optimism for the future.

### Coping

Participants reported a variety of strategies that helped them destress and cope with the situation, with a focus on managing adverse emotions (see Table 2 for illustrative participant quotes).

#### Problem-focused coping

Almost all participants took precautions to minimize their own infection risk and inadvertent infection of family and friends. These included strict adherence to infection prevention measures, such as masking and handwashing at work and at home to the extent material was available. Many also tried to keep a distance from family, where possible, by sleeping in a different room, not sharing food and not hugging or kissing, but acknowledged that this was often difficult if not impossible. Some health workers even temporarily moved out of their houses. In The Gambia, government provided hotel accommodation to staff working in the biggest COVID-19 treatment centre.

Particularly in the early days, before systematic training on COVID-19 and infection prevention, many participants took an active approach to educating themselves, using the internet as well as colleagues with a background in infectious diseases or who had been trained during Ebola times.

Some participants pursued active health and stress management to fortify themselves physically and psychologically. This included regular physical exercise, sufficient sleep and rest, healthy eating and applying relaxation and other techniques taught by the psychological support services where available.

Several participants further reported considering not coming to work in the early days. A few explicitly admitted to ‘escaping’ the problem by minimizing the time spent with patients or avoiding them altogether.

#### Emotion-focused coping

Most participants spoke of social support as their primary coping mechanism, describing how speaking about their experiences and feelings with the team, their family and friends and the psychological support teams available in some hospitals helped them process and deal with difficulties and frustrations.

Many participants also spoke about how they drew strength from their faith and prayer and about how deferring protection of their well-being to God helped them overcome difficult moments.

Other common emotion-focused coping strategies included escapist strategies such as distracting oneself from thinking about one’s situation, by watching movies, socializing or throwing oneself into work so as to eliminate room for reflection; and focusing one’s thoughts onto optimistic evaluations of the future.

#### Meaning-focused coping

In the early days of the pandemic, many participants quickly resigned to the fact that if they were to honour their professional vows, their lives would be difficult for some time. Against this realization, many described trying to find meaning and purpose in an overall adverse situation. Strategies included frequently reminding oneself of why one had chosen the profession and what one had sworn to when joining the profession, even in the face of adversity; turning to one’s faith, reminding oneself that God would not allow the situation without good reason; and focusing on other positive aspects such as service to the country or the learning opportunities associated with the crisis.

#### Future-oriented coping

A handful of participants reported actively preparing themselves, even before the pandemic hit their work settings, and how this helped to put them more at ease in the face of COVID-19. This included trying to gain as much information and skill as possible as well as mental preparation. Reports of *ex-ante* preparation came predominantly from participants in The Gambia, where the first surge in COVID-19 cases occurred comparatively late.

### Emotions

Narratives converged on seven main emotional experiences during the first nine months of the pandemic resulting from various challenges and attempts at coping with them, which changed as time progressed (see Table 3 for illustrative participant quotes). In describing these in more detail in the following, we highlight heterogeneity where we identified any but, in the interest of brevity, do not point out where no clear patterns emerged.

**Table 3.** Illustrative participant quotes—emotion s

Theme	Illustrative quote(s)
Fear of own infection	The first days were very stressful as we didn’t really know how the illness transmitted. […] This made patient care very very difficult. Our psychological state … a patient needs you, you go to them because you don’t have a choice, it’s your job. But psychologically, you are not prepared, and the [protective] material is not of good quality. It was stressful every second. […] Now, in terms of protection, we feel more at ease (male nurse, COVID-19, Burkina Faso). When I was called to tell me that I was requisitioned for COVID-19 triage, I didn’t sleep. I asked [my supervisor] what I did to get him to give my name. He told me that he just chose at random and reassured us there will be measures so that we are really safe. […] But when they [later] told us that there were now cases in the hospital, it really scared me! I was really, really scared. Now it’s ok, I’m afraid but not like at the very beginning (female nurse, COVID-19, Burkina Faso). We were not trained, we were not given proper equipment. If we had suspected cases, the only thing we used were our mask, and proper handwashing, but other materials we didn’t have. […] Before [COVID-19] started, it was normal … you do see tuberculosis cases in pregnant woman, there are all sorts of diseases that you can get from the patient. But the fear of COVID-19 is too much, different from the others (female nurse, maternity, The Gambia). No, I didn’t feel any worries. I sometimes even tell my colleagues that the safest place is to be in the lab because you are in full PPE and you know exactly what you are dealing with, so you are very careful. Not like when you are out there in the public, when you don’t know who is infected and who is not (male biologist, laboratory, The Gambia). Yesterday somebody said to me ‘I looked at the television, we have 20 or 25 cases, so I am going to remove my mask’. People are completely slacking off. That’s why I’m afraid. And with Tabaski starting soon, I am afraid. I was a little optimistic, but with the religious ceremonies coming up, the virus may return. I don’t know what will follow (female midwife, maternity, Senegal).
Fear of being a risk to others	I don’t want to take the disease and transmit the virus to my family; my biggest worry was that (male nurse, COVID-19, The Gambia). I was quarantined because my mask fell off while I was in the patient area. I developed a fever, and they came to my house to take a sample. It was at this precise moment that I understood that I was a danger for my family and my relatives. Fortunately, I was negative. I moved to find a house where I am alone (male doctor, COVID-19, Burkina Faso).
Guilt	The first days were not easy at all, especially towards my family. I felt guilty, to go and expose myself [at work], and to come back home to be in contact with them. So when I got home, I tended to sit by myself, wear a surgical mask, not wanting them to come near me. With time this anxiety has dissipated, I won’t say totally, but now I feel at ease, it has become a routine for me (female doctor, COVID-19, Burkina Faso).
Compassion	The families are devastated, when someone is sick and you cannot have access to that person, I feel their pain. […] Some of the [patients] are also not taking it easy, the last one we had was so difficult, we had to moved him to the open ward. He was alone in the side room, but there is a glass wall so he was able to see all the nurses and we also able to see him. […] It is very difficult, we actually feel their pain (female nurse, COVID-19, The Gambia).
Anxiety vs optimism regarding the future	I am optimistic, but also anxious because when I go out or when I watch TV, and see how our population behaves, I realize that we [health workers] make efforts to treat those who are ill, but people really don’t respect prevention measures. It’s, excuse my language, like they don’t give a damn (male doctor, emergency, Burkina Faso).
Feeling left alone vs well supported	Towards governments and institutions, particularly emergency and maternity departments: At the beginning of the pandemic we were receiving patients, sometimes there were signs of COVID-19. […] We sounded the alarm and tried to talk to the head of the department, the director, everybody. They told us that it’s paranoia, we’re giving into the *psychosis*^a^. There was no preparation. The first confirmed patients went through the emergency room, many doctors took care of them without protection, there was a lot of contamination. I was furious, I think it was negligence. We could have been prepared if we had wanted to, since January we talked about it, but we were not listened to (female doctor, emergency, Burkina Faso). Towards direct managers: Doctor X meets with us, it cheers us up that our superiors really think a lot about us. […] We discuss [our issues], each one gives their ideas. There is much [support] with the superiors, especially with Doctor X (male nurse, emergency, Senegal). Towards team members: We really feel the presence of a team, of a very positive team spirit. Religions, ethnicities, other things, we don’t see the difference. […] Sometimes we pay for a snack for everyone, or we make tea for everyone, to create a positive team spirit, to support each other, because we don’t want to isolate ourselves, because we are also isolated (male hygienist, COVID-19, Senegal).
Feeling unappreciated, devalued and disrespected vs appreciated, valued and respected	Towards governments and institutions: It’s as if we are cannon fodder. […] Why don’t they give us enough protection? (Male doctor, emergency, Senegal) I think that after COVID-19, a lot of doctors will throw in the towel (male doctor, COVID-19, Senegal). We’re not taken into account. There were billions and billions in [the COVID-19 response]. We don’t know at all [what happened to the money]. All this is revolting. We have the impression of wearing ourselves out for very little. You do the maths, you wonder why you are doing it at all (female doctor, emergency, Burkina Faso).
	Towards patients and wider public: With the patients we feel at ease, it is they who give us the strength to continue with their prayers, their encouragement (male nurse, COVID-19, Senegal). It’s revolting, knowing that you risk your life so that this disease doesn’t spread, people allow themselves to say anything. Really, it’s disappointing (male nurse, COVID-19, Burkina Faso).

a Several French-speaking respondents spoke about ‘la psychose’ when referring to widespread sentiments in the early days of the pandemic. The term is not meant to refer to the respective psychiatric syndrome.

#### Fear of own infection

Fears of infection were described by almost all participants, particularly in the initial weeks, fuelled by a sense of helplessness towards how to protect oneself from catching this new disease. Fears were often linked to insufficient PPE and lack of knowledge and infection prevention routine. Over time, accordingly, fears gradually declined as more became known about the virus and how to handle it, infection prevention material became more readily available and/or more appropriate and health workers developed routines for using it. Significant rates of infection among health workers and within their social networks also contributed to diminishing fears; many reported how they had seen colleagues, family and friends emerge from infection without severe consequences.

Across countries, the magnitude of both initial fears of infection and the rate of decline varied with the place of work. Among COVID-19 staff, many reported high initial fears due to certainty of exposure and the novelty of the situation, while many others stated that their levels of fear were low from the start. Participant narratives suggest two reasons: first, it appears that across countries, priority in both training staff and dispensing scarce PPE was given to COVID-19-treating services, so that COVID-19 staff tended to report comparatively good infection prevention from the onset. Second, COVID-19 treatment teams were composed of health workers who had been chosen due to their specialization and/or experience in infectious diseases, health workers who volunteered to participate in the pandemic response and health workers without relevant background who had been posted to COVID-19 teams by directive. While the former two categories of COVID-19 staff tended to report relatively low levels of fear, disappearing quickly, staff from the latter category tended to express comparatively high levels of fear, which tended to linger longer, although with some exceptions to the rule.

Participants from the emergency and maternity departments, in contrast, described how a comparatively late arrival of adequate PPE and training coupled with uncertainty around patients’ COVID-19 status led to fears of infection that tended to be stronger and longer-lasting than in COVID-19 departments.

Laboratory staff across countries reported feeling very well protected and suffering little to no fear of infection at work as they were used to working with infectious material and PPE was sufficiently available.

A few months into the pandemic, most participants stated that fears of catching the virus themselves were low but had not fully disappeared due to widespread disregard of infection prevention measures in the population and recurrent stockouts of infection prevention material. Accounts of persisting fears also reflected differences in the development of the pandemic across countries. Many participants described how fears corresponded to the current state of transmission and how they flared up in the wake of events potentially propagating infection, such as lifting of government measures or religious festivals, and whenever co-workers, relatives and friends tested positive.

#### Fear of being a risk to others

Closely linked to fears of own infection, many participants spoke about their fears of inadvertently passing the virus on to family members or patients. Concerns about being a risk to the family were more prevalent among participants who lived with their families as opposed to those who did not because they worked in a different location or had moved to temporary accommodation to protect their families.

#### Guilt

Many participants expressed feelings of guilt about potentially exposing their family members to infection, explaining that unlike themselves, their family and friends had not signed up to the health-care profession with all its associated risks. Some participants expressed similar feelings of guilt towards non-COVID-19 patients. Participants, particularly with children too small to understand the circumstances, also expressed guilt over not being able to interact as freely as before due to infection prevention measures and about not being able to spend as much time with family and friends due to long work hours.

#### Compassion

Among participants from COVID-19 departments, many spoke about the unusually close emotional bond they formed with patients and their families and how this led to previously unknown compassion and second-hand suffering. They mentioned, in particular, patients being alone with their fears and anxieties, missing their loved ones, in stark contrast to normal practice where family members provide emotional care during hospital stays; and family members distraught over not being allowed to collect bodies of deceased relatives in the early days of the pandemic.

#### Anxiety vs optimism regarding the future

Most participants expressed alternating and sometimes also coexisting bouts of anxiety and optimism about how the situation would unfold. While optimism developed in times of declining case numbers, anxieties stemmed from widespread misbelief within populations in all three countries leading to disrespect of pandemic control measures; perceived inability of the authorities to put into place and enforce necessary measures; mistrust of official case numbers, particularly in Senegal where several health workers reported having witnessed deaths likely due to COVID-19, but not registered as such; and more generally from the under-resourced and insufficiently prepared state of their countries’ health systems. Beyond the health impact, participants were anxious about what this would mean for their countries’ economy as well as for health workers themselves.

#### Feeling left alone vs well supported

Narratives paint a complex picture as to how participants felt supported, technically and emotionally. Most felt abandoned in dealing with the emerging crisis by their governments, ministries of health and institutions, with untimely or inadequate training, shortages in PPE and resources to treat patients and generally unpleasant working conditions. Participants described how pleas and negotiations for improvement were usually in vain, leaving them helpless in difficult situations. Although cognizant of the limitations of health systems, participants complained consistently about poor communication and inaction. This was particularly true for the government institutions, while participants from the two private institutions spoke largely positively about the institutional support received and conditions provided. There was also a striking difference between participants from COVID-19 departments and laboratories vs maternity and emergency departments. Whereas participants from the former reported feeling comparatively well taken care off, particularly in terms of PPE, the opposite was true for the latter where many health workers across countries and institutions reported feeling forgotten and neglected.

Over time, participants perceived some improvements in institutional support, but more so in COVID-19 departments than in maternity and emergency departments. The difference in experiences might be explained by the fact that in the larger COVID-19 departments, psychological support teams were deployed to help staff and patients deal with the difficult circumstances, which was highly appreciated. In contrast, several participants from smaller COVID-19 treatment centres as well as from other departments had to plead for similar support.

With regard to direct managers, most participants described fantastic leadership, emotional support and encouragement. Participants also praised efforts to make resources available, while acknowledging that scope of action in this regard was often limited.

With the notable exception of one hospital, almost all participants felt well supported by their team members, in terms of sharing the workload as well as emotionally. In fact, one of the strongest themes overall was that of work teams growing together in the face of adversity. Participants particularly from the COVID-19 departments described how they quickly and deeply bonded with their new colleagues. They explained that in a situation where family and friends had difficulty understanding what they were going through, they felt cared for, supported and motivated by their colleagues. These strong bonds, often transcending hierarchies as seemed impossible pre-pandemic, were highlighted as one of the main positive aspects of the pandemic.

Finally, participants reported diverse experiences regarding support from families and friends. Most experienced their personal network as the main pillar of support, but some lived through the opposite, experiencing pressure to quit and other opposition, particularly in the early days of the pandemic when little was known about the virus and its risks.

#### Feeling unappreciated, devalued and disrespected vs appreciated, valued and respected

Closely related to the above, participants described mixed feelings about the extent to which they were valued and appreciated for their efforts, risk-taking and sacrifices in caring for patients during the pandemic.

Many participants took the lack of technical preparation and support received by their employers and governments as well as the unfavourable working conditions as a signal of disrespect and lack of valuation. Several participants across the three countries stated considering employment outside the public health sector in response to their perceived maltreatment during the pandemic.

Feelings of disrespect and lack of valuation were amplified by two issues: first, selection into the COVID-19 treatment teams varied from setting to setting, but a substantial number of participants did not have any option of declining. Many affected were troubled with making sense of what they had done to deserve this ‘punishment’. Second, participants across countries noted that pandemic-specific bonus payments or hardship allowances were promised to some health workers, but not to others. Narratives as to who was eligible did not paint clear pictures, suggesting that participants themselves experienced little transparency. Of those who knew they would receive bonus payments, many spoke of gross delays, turning the bonuses into demotivators rather than tokens of appreciation. Those not eligible expressed strong feelings of unfairness, particularly since the promise of additional remuneration was an important motivator to join the COVID-19 response in the first place. Irrespective of whether eligible or not, participants experienced the governments’ handling of these financial aspects as grossly disrespectful to them, showing a lack of appreciation for their effort and risk-taking.

There was some variation, by country, whether participants felt appreciated for their efforts by their patients, families and the wider public. In Senegal, participants spoke positively about how they were appreciated through prayers, gifts, messages and other tokens and how this had helped staying motivated. Several also spoke about a general new-found level of appreciation of the health workforce that made them feel valued and proud.

In Burkina Faso, in contrast, participants were hurt by what they felt were untruthful social media reports about mistreatment of patients in COVID-19 departments, seriously affecting morale and well-being, particularly in the first months of the pandemic. These seemed grounded in suboptimal treatment conditions initially, when hospitals and health workers were not yet set up for adequate service provision, and then propagated on social media by patient family members in ways that only partially corresponded to reality. Following several such incidents, feelings of appreciation from patients and partially also from patient family members improved, turning into a source of motivation and energy for many participants.

Reports from Gambian participants were mixed, with some feeling appreciated by their patients and the public and others not, without clearly discernible patterns.

In all three countries, participants reported being initially shunned by people who feared infection through close contact. Almost all expressed understanding for friends and family who lacked knowledge of the situation, but many were deeply hurt by such behaviour from fellow health workers who they felt should know better, taking it as a sign of disrespect and devaluation of their service. With time and better understanding of the disease and its transmission, relationships largely normalized, although hurt feelings lingered and several participants described incidents of peer stigmatization multiple months into the pandemic.

## Discussion

Our study captured the lived experiences of health workers in Burkina Faso, Senegal and The Gambia in the early months of the pandemic. Although much research on health worker experiences and psychosocial impacts of COVID-19 was conducted globally, our study fills an important gap in evidence as research from SSA is lacking. It is also an important methodological contribution, demonstrating the feasibility and value of conducting qualitative research remotely in settings where face-to-face contact was, to date, deemed inevitable for high-quality data.

**Figure 2.**
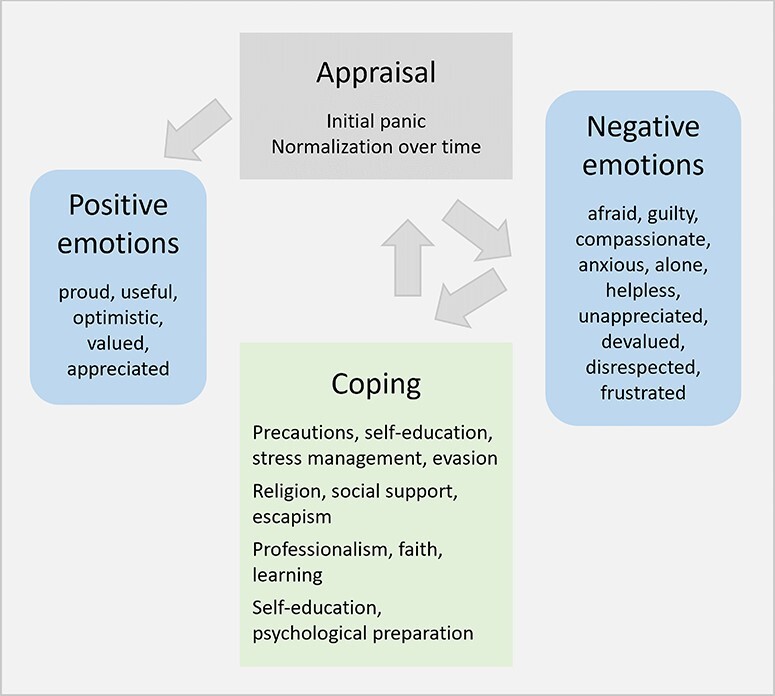
Summary of results

Our findings, summarized in Figure 2, largely mirror what health workers in other parts of the world experienced in the early days of the pandemic (Agyei *et al.*, 2021; Billings *et al.*, 2021; Chemali *et al.*, 2022). Despite notable differences in preparedness, resources and caseload across settings, narratives converge in that the first months of the pandemic were a period of extreme stress, fear and anxiety as health workers struggled through the unknown. Unlike what the literature on health worker experiences in SSA available to date suggested, but in line with the wider international literature (Agyei *et al.*, 2021), negative emotions were gradually complemented by positive feelings of pride, purpose and appreciation, so that our study participants seem to have emerged psychologically relatively unharmed from the first months of the pandemic.

Reasons certainly include that our study sites did not experience significant patient overload. Furthermore, our participants represent a small cohort of the health workforce—highly skilled health workers in secondary and tertiary care hospitals as well as laboratories. Beyond these caveats, however, we are not surprised by the findings. For health workers in resource-limited settings, improvising in the face of uncertainty and lack of resources as well as losing patients to avoidable causes is sadly a frequent reality. As such, we expected a higher level of resilience to adversity compared with health workers in better resourced settings (Wurie and Lohmann, 2020). Future reviews may want to explore experiences and emotional impact of the pandemic in more detail through lenses such as the level of care, place of work and health system and health worker resilience, as also recommended by Chemali *et al.* (2022).

In projecting conclusions beyond the initial year of the pandemic, it is important to note the changing nature of stressors over time. Specifically, appraisals of pandemic threat initially focused on the virus and its direct consequences, gradually turning more positive with the application of various coping strategies, indicating rapid mastery and positive resolution. Over time, however, challenges relating to the management and communication of the pandemic response began to dominate, similar to experiences with previous infectious disease outbreaks (Billings et al., 2021). Participants were particularly frustrated with the fact that many of these challenges could have been avoided with better preparedness, communication and management, such as transparency in staffing decisions and resource distribution, supportive leadership, provisions to protect health worker families and certain basic provisions at the workplace. The longer-term impact of the pandemic on health workers’ psychological well-being will therefore have greatly depended on the resolution of these challenges.

With a view towards future emergency preparedness, we highlight the following: first, in the absence of substantial institutional support, health workers primarily relied on personal coping resources, particularly in the early days, again resonating with the international literature (Agyei *et al.*, 2021; Sehularo *et al.*, 2021) and previous crises (Witter *et al.*, 2017). Although social support from immediate supervisors and work teams was described as important, this tended to be tied to individual relationships and competencies rather than systemic pillars. Many participants lauded the professional psychological support eventually provided in some hospitals and welcomed offers of temporary accommodation outside family homes, underlining the value of structured institutional support. Second, beyond core infection prevention measures, only few participants described proactive or even anticipatory coping approaches aimed at preventing or alleviating the stressful circumstances. Rather, most participants relied on reactive coping strategies to manage adverse emotional experiences.

Research on the relative effectiveness of various coping strategies in protecting mental and physical health paints a complex picture, highlighting dependencies on context and culture, the individual and the nature of the stressful situation, such as its level of controllability and whether it is acute or chronic (Aldwin *et al.*, 2018; Folkman and Moskowitz, 2004). Emotion-focused strategies can play an important role in acute crises or when there is very limited control over the stressful situation, but strategies focused on changing the stressful circumstances and preventing adverse emotional impact become preferable as the stressful situation transitions from acute to chronic, as the pandemic arguably did over the course of 2020. While we did not systematically ask participants about the reasons and purposiveness with which they employed certain coping strategies, we note that almost all could draw on excellent emotion-focused coping resources. To supplement these, it will be of value to systematically strengthen health workers’ proactive and anticipatory coping resources in preparation for future crises.

As health systems are starting to prepare for future similar scenarios, our study therefore suggests the value of the following to preserve health workers’ well-being and strengthen resilience: (1) establish communication strategies and channels that give voice to all to avoid sentiments of perceived unfairness, intransparency and helplessness; (2) implement institutional support measures, including psychological support, policies and mechanisms to protect health workers’ families and training in interpersonal leadership; and (3) promote interventions to foster health workers’ psychological preparedness, coping resources and resilience more generally.

Health worker-focused interventions to foster resilience, prevent occupational stress and train supervisors in human resource management have been reviewed in several Cochrane reviews prior to the pandemic (Ruotsalainen *et al*., 2015; Kuehnl *et al.*, 2019; Kunzler *et al.*, 2020; Pollock *et al.*, 2020). All concluded that the evidence base did not yet allow tangible recommendations, particularly outside well-resourced settings. Research on interventions developed or tested in the context of the pandemic is slowly emerging also from non-high-income settings (e.g. Zingela *et al.*, 2022). Based on our findings, policymakers in Burkina Faso, Senegal and The Gambia will want to be on a particular lookout for tested interventions that strengthen anticipatory coping skills and that can realistically be offered to the entire health workforce, not only to those on the very frontline of a particular disaster.

### Methodological considerations

Our study’s longitudinal nature sets it apart from the existing body of literature on health worker experiences of the pandemic, which is based almost exclusively on one-time data collection. Over time, study participants were increasingly open to speaking about their feelings, highlighting the value of longitudinal research, although motivation to regularly share experiences was impacted by their workload and declined as the situation normalized. The study further demonstrates the feasibility and value of conducting high-quality qualitative research remotely. Remote data collection is an important new methodological tool, as face-to-face data collection is not only time-intensive and costly but also often impossible in many settings due to accessibility challenges.

Our study also has limitations. We relied on volunteers because purposive sampling was deemed unrealistic given the immense pressure health workers were under in the early days of the pandemic. Although key informants’ narratives mirrored those of our participants, we cannot exclude biases introduced through this approach. We conducted the research only in secondary- and tertiary-level health facilities, and primarily with clinical, skilled health-care personnel. Our findings may therefore not generalize to community and primary level or lesser skilled health workers. Finally, although we did not perceive virtual interviewing to have adversely impacted the quality or depth of data obtained, we cannot exclude omission or downplay of certain emotions or coping strategies, particularly socially undesirable ones. For instance, none of our participants spoke about substance abuse, either personal or among colleagues, and very few spoke about impacts on quality of care as health workers attempted to avoid infection.

## Conclusion

Health workers in Burkina Faso, Senegal and The Gambia experienced the first months of the pandemic as a time of extreme stress, characterized by fear for themselves and their loved ones, feelings of isolation and helplessness and perceived lack of valuation. Yet, narratives also demonstrate the remarkable resilience of the health workforce. Employing a wide variety of coping strategies, based mainly on personal resources rather than institutional support, the vast majority managed to weather the first months without severe mental health consequences. Our findings underline the value of participation and transparent communication, of institutional support, and of routine training to foster health workers’ psychological preparedness, coping skill set and resilience more generally.

## Data Availability

The data underlying this article cannot be shared publicly as consent was not obtained. For the purpose of quality control, data will be shared on reasonable request to the corresponding author.

## Reflexivity statement

As was likely the case with most COVID-19-related research, we planned and conducted the research ad hoc, without additional dedicated human resources, within the framework of a prior project on health worker well-being led by the first author. The slight imbalances in gender and the rather senior author team reflect this ad hoc nature of the research. The team includes researchers who had time and capacity for and interest in collaborating in the issue—this included funded positions allowing for taking on such ad hoc work, as well as local networks to conduct the research in the difficult early months of COVID-19. Three of the authors (M.D., J.-L.K. and M.M.-A.) are and were physically based in the study countries at the time of research, while J.L. and M.D.A. have over a decade of research experience in Burkina Faso, including extensive time spent in country.
